# Genetic profiling and diagnostic strategies for patients with ectodermal dysplasias in Korea

**DOI:** 10.1186/s13023-024-03331-6

**Published:** 2024-09-07

**Authors:** Man Jin Kim, Jee-Soo Lee, Seung Won Chae, Sung Im Cho, Jangsup Moon, Jung Min Ko, Jong-Hee Chae, Moon-Woo Seong

**Affiliations:** 1 Department of Genomic Medicine, Seoul National University Hospital, Seoul, Korea; 2 Department of Laboratory Medicine, Seoul National University Hospital, 101, Daehak-ro, Jongno-gu, Seoul, 110-744 Korea; 3 Cancer Research Institute, Seoul National University College of Medicine, Seoul, Korea; 4 Department of Pediatrics, Seoul National University Hospital, Seoul National University College of Medicine, Seoul, Korea

**Keywords:** Ectodermal dysplasia, Genetic diseases, Inborn, High-throughput nucleotide sequencing, Exome sequencing, Korea

## Abstract

**Background:**

Ectodermal dysplasia (ED) is a rare genetic disorder that affects structures derived from the ectodermal germ layer.

**Results:**

In this study, we analyzed the genetic profiles of 27 Korean patients with ED. Whole exome sequencing (WES) was performed on 23 patients, and targeted panel sequencing was conducted on the remaining 4 patients. Among the patients in the cohort, 74.1% (20/27) tested positive for ED. Of these positive cases, *EDA* and *EDAR* mutations were found in 80% (16/20). Notably, 23.1% (3/13) of *EDA*-positive cases exhibited copy number variations. Among the 23 patients who underwent WES, we conducted a virtual panel analysis of eight well-known genes, resulting in diagnoses for 56.5% (13/23) of the cases. Additionally, further analysis of approximately 5,000 OMIM genes identified four more cases, increasing the overall positivity rate by approximately 17%. These findings underscore the potential of WES for improving the diagnostic yield of ED. Remarkably, 94.1% of the patients manifesting the complete triad of ED symptoms (hair/skin/dental) displayed detectable *EDA/EDAR* mutations. In contrast, none of the 7 patients without these three symptoms exhibited *EDA/EDAR* mutations.

**Conclusions:**

When conducting molecular diagnostics for ED, opting for targeted sequencing of *EDA/EDAR* mutations is advisable for cases with classical symptoms, while WES is deemed an effective strategy for cases in which these symptoms are absent.

**Supplementary Information:**

The online version contains supplementary material available at 10.1186/s13023-024-03331-6.

## Background

Historically, conditions classified as ectodermal dysplasia (ED) have exhibited genetic and clinical heterogeneity. A unifying factor among these conditions is the genetic basis of developmental anomalies in tissues originating from the ectoderm [1]. The ectoderm gives rise to various structures, including the epidermis, central and peripheral nervous systems, placodes (including cranial placodes), and neural crest cells [2]. Early classification systems, such as those formulated by Dr. Newton Freire-Maia, categorized EDs based on their inheritance patterns and phenotypic characteristics. The Freire-Maia classification has significantly contributed to our understanding of these disorders and directed approaches to their management. The conditions were classified into two groups based on the extent of tissue involvement: group A, characterized by cases where at least two critical ectodermal tissues, such as hair, teeth, nails, eccrine and other glands, were affected, and group B, encompassing conditions that involved one of the aforementioned tissues along with at least one other ectodermal derivative [2].

Advancements in our understanding of the human genome have revealed molecular correlations among certain ED conditions beyond their traditional categorization based on phenotypes. The identification of causative genetic mutations in genes such as *EDA*,* EDAR*,* EDARADD*,* TRAF6*, and NFkB pathway-related genes has provided insights into the genetic foundation of EDs [3, 4]. These genetic mutations can yield comparable clinical phenotypes, and heterozygosity, compound heterozygosity, and homozygosity can contribute to these disorders. Almost 50% of historically classified EDs are now recognized to have causative genetic mutations encompassing those underlying more common ED conditions. Over the last decade, significant developments have been made in molecular methodologies that facilitate diagnostic differentiation for EDs [5]. Given the expanding therapeutic possibilities, these diagnoses have significant clinical value. Therefore, it is crucial to establish effective strategies for molecular diagnosis tailored to each patient. The choice between targeted panel sequencing (TPS) and whole exome sequencing (WES) should be guided by specific phenotypes observed in ED cases, emphasizing the need for a well-defined strategy.

## Materials and methods

### Patients and enrolment criteria

We enrolled 27 patients with ED between August 2018 and October 2022. We obtained 15 samples from 11 clinical sites throughout South Korea, which were sent to the coordinating center of the Korean Genetic Diagnosis Program for Rare Diseases (KGDP) Phase II, Molecular Diagnostics Laboratory, Seoul National University Hospital (SNUH) (https://www.ncbi.nlm.nih.gov/gtr/labs/320228/). The remaining 12 samples were obtained from patients with ED who were seen at the Seoul National University Hospital. The inclusion criteria encompassed the enrollment of individuals manifesting at least one of the symptoms associated with ectodermal dysplasia. All genetic tests were conducted at the Molecular Diagnostics Laboratory, and the results were interpreted by clinical pathologists with expertise in molecular diagnostics. All study procedures were approved by the Institutional Review Board of SNUH (IRB number: 2212-114-1389), and the study adhered to the Declaration of Helsinki for biomedical research involving human subjects.

### Workflow of the mutation screening

Among the 27 patients, all 15 patients enrolled by KGDP underwent WES. Additionally, among the remaining 12 patients, those referred before September 2021 underwent TPS, while those referred after October 2021 underwent WES. Furthermore, two patients (ED4, ED26) who showed copy number variations (CNVs) by bioinformatics tool for global normalization (as suggested by NextGENe; SoftGenetics) underwent multiplex ligation-dependent probe amplification (MLPA).

### Targeted panel sequencing (TPS)

Of the 27 patients, 4 (14.8%) underwent TPS to analyze representative genes (*EDA*,* EDAR*,* EDARADD*,* LTBP3*,* MSX1*,* NFKBIA*,* PAX9*,* WNT10A*) associated with ED. TPS was performed as described below. DNA was extracted from peripheral blood using a Chemagic 360 instrument (Perkin Elmer, Baesweiler, Germany). Library preparation was performed according to the SureSelect QXT target enrichment protocol (Agilent Technologies). Paired-end 150 bp sequencing was performed using the MiSeq Dx platform (Illumina, San Diego, California, USA). The generated sequencing data were then aligned to the human reference genome sequence (GRCh37/hg19), with subsequent identification and annotation of qualified variants using NextGENe V.2.4.0.1 (SoftGenetics). The detected variants were subjected to variant classification in accordance with the American College of Medical Genetics and Genomics (ACMG) guidelines [6]. Furthermore, to predict CNVs from the targeted NGS data, a bioinformatics tool for global normalization (as suggested by NextGENe; SoftGenetics) was employed for the log2 ratio calculation.

### Whole exome sequencing

DNA was isolated from the peripheral blood of the remaining 23 patients (85.2%) for WES using a Chemagic 360 instrument (Perkin Elmer, Baesweiler, Germany). The extracted DNA was fragmented using a Covaris E220 focused ultrasonicator (Covaris, Woburn, MA, USA). DNA fragments were targeted and captured using the Agilent SureSelect All Exon V8 (Agilent Technologies, Santa Clara, CA, USA). A total of 500 ng of genomic DNA was used as the input. Library preparation was performed using the SureSelect XT Target Enrichment Protocol (Agilent Technologies). Paired-end 150 bp sequencing was conducted using the NovaSeq 6000 platform (Illumina). Bioinformatics processes from alignment to annotation were performed using NextGene (Version 2.4.0.1; Software Genetics, State College, PA, USA).

### Multiplex ligation-dependent probe amplification (MLPA)

DNA denaturation, probe hybridization, ligation, and PCR of the ligated probes were conducted in accordance with the manufacturer’s instructions. The amplified products were subsequently analyzed using an ABI 3130xl capillary sequencer (Applied Biosystems, Foster City, CA, USA). The GeneMarker V.1.51 software (SoftGenetics) was employed for the determination of fragment length and copy number for each fragment.

### Variant interpretation

All identified variants were categorized according to the five-tier system delineated by the ACMG, which encompasses pathogenic, likely pathogenic, variants of uncertain significance (VUS), likely benign, and benign classifications. Variants falling into the pathogenic or likely pathogenic categories were deemed clinically significant. Multiple databases, including HGMD (www.hgmd.org), ClinVar (www.ncbi.nlm.nih.gov/clinvar/intro/), and OMIM (https://www.omim.org/), were consulted for reference [6].

### Statistical analysis

Categorical variables were presented as counts and percentages (%) and analyzed using the χ2 test. All statistical analyses were performed using the R V.4.0.0 (The R Foundation; www.r-project.org). Statistical significance was established for p-values less than 0.05.

## Results

### Descriptive analysis of mutation-positive ED cases

Descriptive statistics for the 20 positive cases are presented in Table 1. Hypotrichosis was observed in all but one case (ED16), and anhidrosis/hypohidrosis was present in all but two cases (ED16 and ED23). Hypodontia/anodontia was observed in all cases except three (ED6, 9, and 23). For ED23, accurate tooth assessment is challenging because of delayed dentition. Altogether cases carrying mutations in the EDA and EDAR genes were 16, accounting for 80% (16/20) of the total positive cases. Among these, *EDA* accounted for the majority (13 cases), and notably, CNVs were responsible for three cases (23.1%). The remaining genes in which mutations were detected included one case each of *ERCC2*, *DSG4*, *LRP6*, and *LIPH*.

**Table 1:** Descriptive statistics of positive ectodermal dysplasia cases (*n* = 20)

Pts.	Age at testing (yr)	Sex	Clinical Characteristics			Other phenotypes	Gene	MANE transcript	Disease	MIM	Mode of inheritance	Variant	Classification
			Hypotrichosis	Hypohidrosis	Hypodontia/Anodontia								
ED2	39	M	Yes	Yes	Yes	eczema	*EDA*	NM_001399.5	XLHED	305,100	XLR	c.252del, p.Gly85Alafs*6, hem	P
ED3	20	M	Yes	Yes	Yes		*EDAR*	NM_022336.4	Ectodermal dysplasia 10 A	129,490	AD	c.1043T > C, p.Leu348Pro, het	LP
ED4	1	M	Yes	Yes	Yes	facial asymmetry, hemivertebra, atopic dermatitis	*EDA*	NM_001399.5	XLHED	305,100	XLR	exon 2 deletion, hem	P
ED6	5	M	Yes	Yes	No*	frequent febrile episode, micropenis, adrenal hemorrhage	*ERCC2*	NM_000400.4	Trichothiodystrophy 1, photosensitive	601,675	AR	c.591_594del, p.Tyr197*, het	P
												c.1004G > A, p.Arg335Gln, het	LP
ED7	28	M	Yes	Yes	Yes	depressed nasal dorsum, oriental eyelid	*EDA*	NM_001399.5	XLHED	305,100	XLR	c.572del, p.Pro191Glnfs*89, hem	P
ED9	0^†^	F	Yes	Yes	No	skin tag of anus	*DSG4*	NM_177986.5	Hypotrichosis 6	607,903	AR	c.574T > C, p.Ser192Pro, hom	LP
ED10	3	M	Yes	Yes	Yes		*EDA*	NM_001399.5	XLHED	305,100	XLR	c.599dup, p.Gly201Argfs*39, hem	P
ED11	1	M	Yes	Yes	Yes		*EDA*	NM_001399.5	XLHED	305,100	XLR	c.1142G > A, p.Gly381Glu, hem	LP
ED12	8	M	Yes	Yes	Yes	atopic dermatitis	*EDAR*	NM_022336.4	Ectodermal dysplasia 10 A	129,490	AD	c.1258 C > T, p.Arg420Trp, het	LP
ED13	19	M	Yes	Yes	Yes	atopic dermatitis	*EDA*	NM_001399.5	XLHED	305,100	XLR	c.1049G > A, p.Gly350Asp, hem	LP
ED14	19	M	Yes	Yes	Yes		*EDA*	NM_001399.5	XLHED	305,100	XLR	c.1133 C > T, p.Thr378Met, hem	LP
ED15	1	M	Yes	Yes	Yes		*EDA*	NM_001399.5	XLHED	305,100	XLR	exon 1–2 deletion, hem	P
ED16	4	M	No	No	Yes		*LRP6*	NM_002336.3	Tooth agenesis, selective, 7	616,724	AD	c.94 C > T, p.Arg32*, het	P
ED19	40	M	Yes	Yes	Yes		*EDA*	NM_001399.5	XLHED	305,100	XLR	c.707del, p.Gly236Valfs*44, hem	LP
ED20	17	M	Yes	Yes	Yes	nail dystrophy, atopic dermatitis	*EDA*	NM_001399.5	XLHED	305,100	XLR	c.599dup, p.Gly201Argfs*39, hem	P
ED21	2	M	Yes	Yes	Yes		*EDA*	NM_001399.5	XLHED	305,100	XLR	c.457 C > T, p.Arg153Cys, hem	P
ED23	7	M	Yes	No	NA^‡^		*LIPH*	NM_139248.3	Hypotrichosis 7	604,379	AR	c.736T > A, p.Cys246Ser, het	P
												c.928del, p.Asp310Ilefs*4, het	LP
ED25	1	M	Yes	Yes	Yes		*EDA*	NM_001399.5	XLHED	305,100	XLR	c.463 C > T, p.Arg155Cys, hem	P
ED26	32	M	Yes	Yes	Yes		*EDA*	NM_001399.5	XLHED	305,100	XLR	exon 2 duplication, hem	P
ED27	17	F	Yes	Yes	Yes		*EDAR*	NM_022336.4	Ectodermal dysplasia 10 A	129,490	AD	c.1259G > A, p.Arg420Gln, het	LP

* Dystrophic teeth are observed

† 3 days

‡ Not assessed due to delayed dentition

XLHED: X-linked hypohidrotic ectodermal dysplasia; XLR: X-linked recessive; AD: autosomal dominant; AR: autosomal recessive; NA: not assessed; hem: hemizygote; Het: heterozygote; P: pathogenic; LP: likely pathogenic

### Cases with EDA/EDAR mutations

**Fig. 1:**
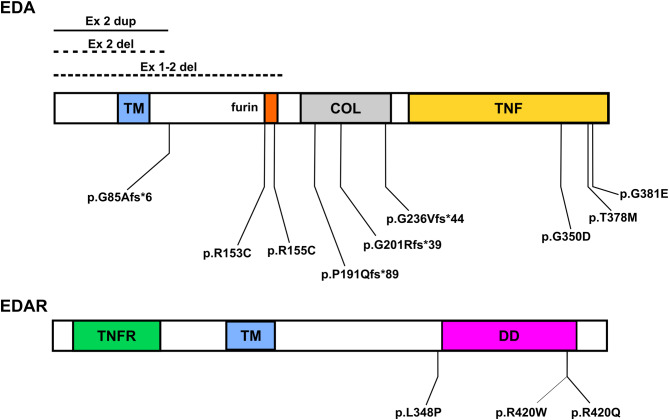
Genetic variants of *EDA/EDAR* in positive ectodermal dysplasia cases. Among 16 cases, 15 sequence variants were identified (p.G201Rfs*39 was detected in both ED10 and ED20). Three EDA CNVs are shown at the top left. Among the four frameshift mutations, three were located in the COL domain of the EDA gene, two missense mutations were in the furin domain, and three were at the end of the TNF domain. Three missense *EDAR* mutations were identified: two in the C-terminal end of the DD and one slightly upstream of the DD domain. COL, collagen-like domain; TNF, tumor necrosis factor; DD, death domain

*EDA* and *EDAR* mutations were detected in 16 cases, including 15 male patients and 1 female patient, who tested positive for *EDAR*. The average patient age was 15.5 years. In positive cases, mutation sites within *EDA* and *EDAR* were annotated in the functional domains. (Fig. 1). Missense mutations were localized toward the termini of the established furin and TNF domains, whereas loss-of-function (LOF) mutations were predominantly clustered within the COL domain. Concerning *EDAR* mutations, three alterations were detected in the death domain. Notably, a novel missense mutation in *EDAR* gene (NM_022336 c.1043T > C, p.L348P) was confirmed as a de novo mutation based on parental testing results (Supplementary Figure S1).

### Cases with mutations in genes other than *EDA/EDAR*

ED6 had biallelic mutations on *ERCC2.* He is a 5-year-old boy who exhibited classic symptoms including hypotrichosis and hypohidrosis. Hypodontia was not prominent; instead, dysmorphic teeth were observed. Notably, he had a history of frequent febrile episodes, along with the presence of micropenis accompanied by underdeveloped scrotum, and adrenal hemorrhage during the neonatal period (Table 1). *ERCC2* is associated with xeroderma pigmentosum group D (MIM: 278730) in an autosomal recessive manner. Moreover, biallelic mutations in *ERCC2* can also lead to photosensitive trichothiodystrophy. Two mutations in *ERCC2* were identified: a frameshift mutation and a missense mutation that was initially categorized as VUS. However, subsequent parental testing led to the segregation and reclassification of these two mutations as likely pathogenic, thereby confirming the genetic basis of the disease.

ED9 had a homozygous mutation, c.574T > C, p.Ser192Pro, in the *DSG4* gene. *DSG4* is the causal gene of autosomal recessive hypotrichosis 6 (MIM: 607903), which is inherited in an autosomal recessive manner. Interestingly, ED9 exhibited hypohidrosis, whereas typically *DSG4*-mutation positive patients show normal sweating.

ED16, a 4-year-old boy, presented with a normal complement of 20 deciduous teeth; however, routine X-rays revealed multiple missing permanent teeth (more than half absent). A de novo mutation in *LRP6* was detected. This variant is a heterozygous pathogenic nonsense mutation (c.94 C > T, p.Arg32*) in the *LRP6* gene. *LRP6* is associated with the autosomal dominant tooth agenesis-selective 7 (MIM: 616724).

Lastly, ED23, a 7-year-old boy, exhibited hypotrichosis, although hypohidrosis was not observed. Hypodontia could not be confirmed due to delayed dentition. ED23 patient harbored a pathogenic mutation (c.736T > A, p.Cys246Ser, heterozygous) and a likely pathogenic frameshift variant (c.928del, p.Asp310Ilefs*4, heterozygous) in the *LIPH* gene. *LIPH* is associated with autosomal recessive hypotrichosis 7 (MIM: 604379).

### Analysis of negative cases with ED symptoms

Table 2 displays 7 negative cases. Among these, all seven patients were male, with dental issues evident in six cases (85.7%), hypohidrosis/anhidrosis in two cases (28.6%), and hypotrichosis in three cases (42.9%). Furthermore, three patients exhibited symptoms unrelated to the ectodermal origin, such as optic neuropathy and failure to thrive. Novel VUS were identified in four patients. Specifically, *WNT10A* variants were observed in 2 cases, and *EDA* and *KDF1* were present in 1 case. Except for the *WNT10A* c.511 C > T variant, the remaining three variants were predicted to be deleterious based on all three in silico prediction tools. The sequence variants identified in *EDA* and *KDF1* are not annotated in the GnomAD database of human variations. Parental testing was only feasible for the *KDF1* case, where the mother was found to be a carrier and had few permanent teeth.

**Table 2:** Descriptive statistics of negative ectodermal dysplasia cases (*n* = 7)

Pts.	Age at testing (yr)	Sex	Clinical characteristics			Other phenotypes	Gene	MANE transcript	Disease	MIM	Mode of inheritance	Variant	gnomAD (%)	SIFT	Polyphen	MutationTaster	Classification
			Hair	Skin	Dental												
ED1	10	M	brown colored hair, no eyebrows or eyelashes	normal sweating	normal	optic neuropathy, DD, low set ear, microcephaly, spastic gait											
ED5	7	M	hypotrichosis	normal sweating	hypodontia	failure to thrive, hydrocele											
ED8	11	M	hypotrichosis	anhidrosis	anodontia	nail dystrophy, hypothyroidism, obesity, osteoporosis, lightly pigmented eyes, limping gait	*EDA*	NM_001399.5	XLHED	305,100	XLR	c.410 A > T, p.Asn137Ile, hem	None	Deleterious	Probably damaging	Deleterious	VUS
ED17	9	M	normal	normal	hypodontia												
ED18	12	M	normal	hypohidrosis	hypodontia		*KDF1*	NM_152365.3	Ectodermal dysplasia 12	617,337	AD	c.911T > G, p.Ile304Ser, het	None	Deleterious	Probably damaging	Deleterious	VUS
ED22	7	M	hypotrichosis	NA	hypodontia		*WNT10A*	NM_025216.3	Tooth agenesis, selective, 4	150,400	AD	c.511 C > T, p.Arg171Cys, het	0.19	Deleterious	Probably damaging	Deleterious	VUS
ED24	39	M	normal	normal	hypodontia		*WNT10A*	NM_025216.3	Tooth agenesis, selective, 4	150,400	AD	c.364 A > T, p.Ile122Phe, het	0.004	Deleterious	Benign	Deleterious	VUS

Pts, patients; DD, developmental delay; NA, not assessed; XLHED, X-linked hypohidrotic ectodermal dysplasia; XLR, X-linked recessive; AD, autosomal dominant; AR, autosomal recessive; hem, hemizygote; Het, heterozygote; VUS, variant of uncertain significance

### Correlation between *EDA/EDAR* mutation detection and classic symptom presentation

Table 3 illustrates the mutation detection rates of *EDA* and *EDAR* based on the presence of hair, skin, or dental symptoms. Notably, of patients exhibiting all three symptoms, 94.1% had *EDA/EDAR* mutations; of those who did not show these three symptoms, none had *EDA/EDAR* mutations.

**Table 3:** Detection rate of *EDA/EDAR* mutations according to symptoms

Clinical characteristics	Positive yield in *EDA*, *EDAR* genes	*P*
Hair/ Skin/ Dental		
All present	94.1% (16/17)	< 0.0001
Not all present	0% (0/10)	

## Discussion

To date, this is the largest molecular study conducted in a Korean population with suspected ED. The *EDA* and *EDAR* genes constitute the molecular basis of 74.1% of patients with ED. The detection of mutations in relatively novel genes, including *DSG4*, *ERCC2*, *LIPH*, and *LRP6*, which elucidate disorders characterized by only one or two classic symptoms of ED, underscores the efficacy of WES [7].

The number of genome-wide methods capable of diagnosing rare diseases has been increasing. However, to optimize the allocation of the limited funds available for clinical NGS diagnostics, it is essential to utilize existing resources in an efficient and economically viable manner [8]. Therefore, selecting an appropriate genetic test based on the phenotype of a rare disease is an efficient strategy. We demonstrated the presence of *EDA* and *EDAR* mutations in over 90% of patients exhibiting the three classical ectodermal symptoms.

In the case of one patient with VUS on *EDA*, the c.410 A > T variant was reported as a VUS with “insufficient evidence” in ClinVar. For *EDA* patients, this variant has been previously reported to be absent in the normal population and has been predicted to be deleterious by in silico prediction tools such as Sorting Intolerant From Tolerant (SIFT) [9], Polymorphism Phenotyping version 2 (PolyPhen-2) [10], and Combined Annotation-Dependent Depletion (CADD) [11]. Therefore, there is a possibility that it may be reclassified as likely pathogenic based on additional evidence, such as segregation study data, in the future.

However, in cases where classical ED symptoms are not fully exhibited, or when other tissue symptoms are present, such as in Group B, WES may be a favorable strategy. Furthermore, in cases of atypical ED, various genes have sporadically been observed to have mutations, and new genes continue to be discovered [12, 13]. Conversely, in cases where patients exhibit typical ED symptoms but test negative, techniques such as MLPA or WGS should be considered because of the possibility of mutations that are not easily detected by conventional NGS methods, such as CNVs or structural variations in *EDA* and *EDAR*.

Promising therapeutic avenues have emerged for patients with ED [14]. A drug-targeting strategy involving the neonatal Fc receptor has shown potential in addressing sweating deficiency associated with X-linked hypohidrotic ectodermal dysplasia, especially when administered during fetal development [15, 16]. Ongoing clinical trials of fetal therapy aim to validate the observed enhancements in male fetuses undergoing in utero treatment. Moreover, there are ongoing advancements in translational research on regenerative therapies for skin and corneal lesions using patient-derived stem cells, and dental interventions are being devised to enhance oral function in patients with EDs [5]. Thus, establishing diagnostic strategies for ED is becoming increasingly important. Our study sheds light on effective diagnostic strategies based on ED phenotype.

## Conclusions

In conclusion, when performing molecular diagnostics for ED, it is recommended to choose targeted sequencing of *EDA/EDAR* mutations in cases with classical symptoms. In contrast, WES is considered an effective approach for cases lacking these typical symptoms.

## Electronic supplementary material

Below is the link to the electronic supplementary material.

Supplementary Material 1

## Data Availability

We are unable to provide the requested data due to privacy concerns, and therefore cannot offer the URL. It can be made available upon reasonable request to the corresponding author.

## Abbreviations

ED: Ectodermal dysplasia
WES: Whole exome sequencing
TPS: Targeted panel sequencing
KGDP: Korean Genetic Diagnosis Program for Rare Diseases
SNUH: Seoul National University Hospital
CNVs: Copy number variations
MLPA: Multiplex ligation-dependent probe amplification
ACMG: American College of Medical Genetics and Genomics
VUS: Variants of uncertain significance
LOF: Loss-of-function
SIFT: Sorting Intolerant From Tolerant
PolyPhen-2: Polymorphism Phenotyping version 2
CADD: Combined Annotation-Dependent Depletion
