# Supplementary material for: Genetic profiling and diagnostic strategies for patients with ectodermal dysplasias in Korea
Source: Orphanet J Rare Dis. 2024 Sep 7;19:329. doi: 10.1186/s13023-024-03331-6 (PMC11380769; doi:10.1186/s13023-024-03331-6)
Supplement: Supplementary file 1 — Supplementary Material 1 [file 13023_2024_3331_MOESM1_ESM.docx]

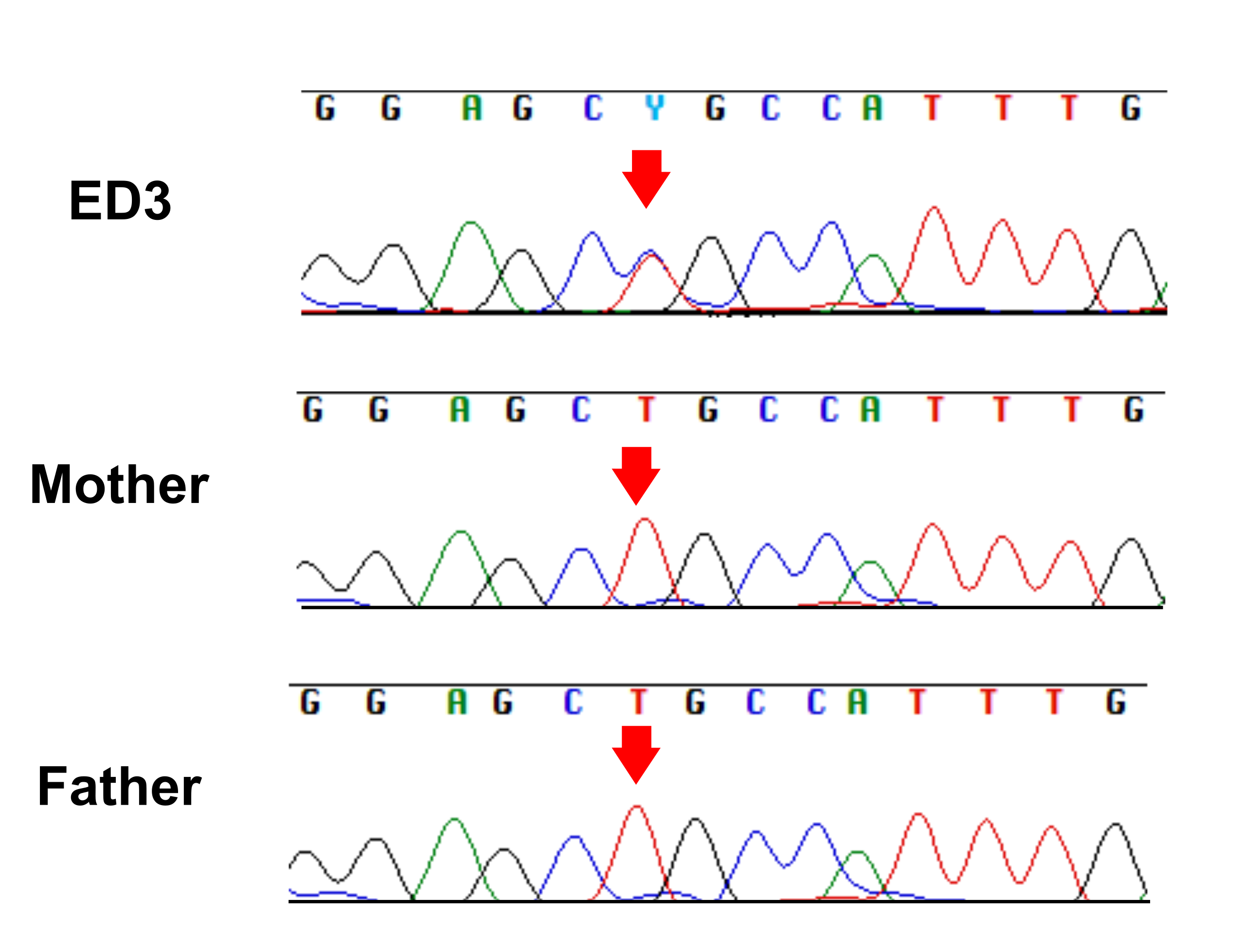


**Figure S1. The trio Sanger sequencing results for *EDAR*, c.1043T>C, p.Leu348Pro, heterozygote of the patient ED3.** The mutation found on ED3 was not detected in his mother and father. Red arrows indicate the variant.
